# Serotonin release from the neuronal cell body and its long-lasting effects on the nervous system

**DOI:** 10.1098/rstb.2014.0196

**Published:** 2015-07-05

**Authors:** Francisco F. De-Miguel, Carolina Leon-Pinzon, Paula Noguez, Bruno Mendez

**Affiliations:** Instituto de Fisiología Celular-Neurociencias, Universidad Nacional Autónoma de México, Circuito Exterior SN, Ciudad Universitaria, Distrito Federal, Mexico

**Keywords:** serotonin, extrasynaptic, somatic exocytosis, transmitter release, neuron–glia communication, serotonergic modulation

## Abstract

Serotonin, a modulator of multiple functions in the nervous system, is released predominantly extrasynaptically from neuronal cell bodies, axons and dendrites. This paper describes how serotonin is released from cell bodies of Retzius neurons in the central nervous system (CNS) of the leech, and how it affects neighbouring glia and neurons. The large Retzius neurons contain serotonin packed in electrodense vesicles. Electrical stimulation with 10 impulses at 1 Hz fails to evoke exocytosis from the cell body, but the same number of impulses at 20 Hz promotes exocytosis via a multistep process. Calcium entry into the neuron triggers calcium-induced calcium release, which activates the transport of vesicle clusters to the plasma membrane. Exocytosis occurs there for several minutes. Serotonin that has been released activates autoreceptors that induce an inositol trisphosphate-dependent calcium increase, which produces further exocytosis. This positive feedback loop subsides when the last vesicles in the cluster fuse and calcium returns to basal levels. Serotonin released from the cell body is taken up by glia and released elsewhere in the CNS. Synchronous bursts of neuronal electrical activity appear minutes later and continue for hours. In this way, a brief train of impulses is translated into a long-term modulation in the nervous system.

## 1. Introduction

Modulation of neural circuits allows a wide variety of state-dependent responses to a particular stimulus. Modulation may last from minutes to months, and may affect responses from simple neuronal circuits that generate reflex responses to other much more complex circuits that produce huge amounts of processing. An example is the performance of a tennis player who must detect the velocity, force and trajectory of a ball approaching at more than 100 km h^−1^. Within hundreds of milliseconds, he must calculate the movement of his body to hit the ball and send it to the opposite extreme of the court. A good tennis player is trained to give very well-controlled reproducible responses. However, these are not at all fixed. Our champion may send the ball out of the court on the day, days or even months that follow the sudden end of a nice romance. How could a brief stimulus change his professional performance for such a long period?

Modulation is not only a source of failures. It is a wider phenomenon that changes the performance of neuronal circuits, from sensory inputs to motor outputs (e.g. [1]). It depends on previous experience and produces integrated changes in the responsiveness of neurons, glia and blood vessels. Transmitter release at synaptic connections may modulate further release. However, at the level of whole neuronal circuits, modulation requires the release of large amounts of signalling molecules from extrasynaptic sites in the soma, dendrites and axons. Molecules released in this way become ingredients of the extracellular ‘broth’ that bathes the nervous system and acts on extrasynaptic receptors, often located distantly from the release sites (for review, see [2–4]).

This paper describes how serotonin is released from the cell body, also called soma, of neurons and produces long-term effects on a population of neurons. We show that a train of electrical impulses at 20 Hz, lasting 0.5 s, triggers a chain of intracellular processes that produce the release of large amounts of serotonin. Each stage expands the timing of the signalling process by one order of magnitude or more. As a result, a brief stimulus is translated into responses that continue for hours.

## 2. Serotonin modulation of the nervous system

Serotonin affects all levels of the function of central and peripheral nervous systems, from sensory neurons to motor outputs, and from development to disease (for review, see [5]). Serotonin acts in every moment of our lives, and failures of our serotonergic system produce depression, bipolar disorder or posttraumatic stress disorder among other syndromes. In newborns, serotonergic failures may cause sudden death [6]. The failures of our favourite tennis player mentioned above may be due to anxiety, a lack of concentration and even depression, with all of these problems involving the serotonergic system.

Several aspects of serotonergic systems of vertebrates and invertebrates are quite striking. As a general rule serotonin is produced by small numbers of neurons. However, most regions of the central nervous system receive serotonergic innervation [7]. The number of synaptic connections formed by these neurons is small [8–11]. Instead, neurons release serotonin from their soma, dendrites and axon varicosities [12–16]. Early studies of serotonergic neurons in the brain also described the presence of extracellular serotonin distant from serotonergic neurons (reviewed in [3]. Other biogenic amines and peptides were found in similar locations, and the poor correlation between the terminals and receptors suggested their release from extrasynaptic sites and their action via volume transmission [3]. It is now well established that serotonin is released extrasynaptically from the soma, dendrites and axon varicosities of vertebrates and invertebrates [13,14,17,18]. The way by which serotonin is released extrasynaptically has been studied in detail using Retzius neurons from the central nervous system of the leech, which have provided most of our direct knowledge about serotonergic synaptic transmission (for review, see [19]).

## 3. Why study somatic release of serotonin in leech?

Invertebrate preparations provide excellent possibilities for the study of basic principles of neuronal functions. Examples for this are the discovery of the ionic basis for the generation of the action potential [20], the demonstration of calcium entry to trigger transmitter release [21], the demonstration that GABA is a neurotransmitter [22], the first studies on the physiology of glial cells [23,24] and the pioneering studies showing that neurons of different function generate cell-type-specific action potential wave forms [25], among many others. Our studies have been carried out using a classical preparation for the study of serotonergic transmission: the Retzius neuron of the leech central nervous system.

A leech central ganglion contains about 400 neurons. Their cell bodies are distributed in a remarkably stereotyped arrangement that allows their visual identification based on the size and position. Most neurons have been identified based on their electrical properties, morphology, the transmitters or peptides they release, their connectivity patterns and their contribution to behaviour [26]. In addition, a glial cell envelopes the neuronal cell bodies [27]. The origin of individual neurons and the steps in the formation of their arborizations have been traced during embryonic development [28]. Moreover, the current molecular biology and genome sequencing tools allow the combination of physiology and developmental studies to understand behaviour [29,30].

The pair of serotonergic Retzius neurons in each ganglion display the largest somata. They incorporate serotonin in clear vesicles at synapses or in electrodense vesicles in the soma, axon and surrounding the synaptic clear vesicles. Adult leech neurons survive in culture for weeks while keeping their identities. Cultured Retzius neurons form serotonergic presynaptic endings onto a pressure sensory (P) neuron [31] or onto another Retzius neuron [32]. Formation of chemical synapses induces redistribution of calcium channels among other adaptive changes [33,34]. Serotonin is synthesized by Retzius neurons and released upon stimulation in quanta from clear or dense-core vesicles [35–37] in a calcium-dependent manner [38].

## 4. Somatic release of serotonin

Serotonin was detected a long time ago inside ‘astronomic numbers' of electrodense vesicles in the soma of Retzius neurons [39]. However, that the vesicles rest distantly from the plasma membrane demolished expectations for a somatic exocytosis. Years later, quantal exocytosis of serotonin from somatic electrodense vesicles was recorded by amperometry in response to a calcium ionophore added to Retzius neurons [40]. This suggested that somatic exocytosis could occur under physiological conditions. It was a decade ago when physiological electrical stimulation in the presence of FM1–43 dye produced a pattern of fluorescent spots in the soma that indicated cycles of calcium-dependent exo/endocytosis as vesicles fuse with the plasma membrane [17]. The dye incorporates into the internal membranes of vesicles that fuse, and therefore fluorescence increases in proportion to the number of vesicles undergoing exocytosis [41]. Since vesicles fuse only once in response to a train of impulses, and their recycling is rather slow [42,43], the kinetics of the formation of fluorescent spots indicates the kinetics of exocytosis, while the number of spots in each soma is a measure of the amount of exocytosis [17].

**Figure 1.**
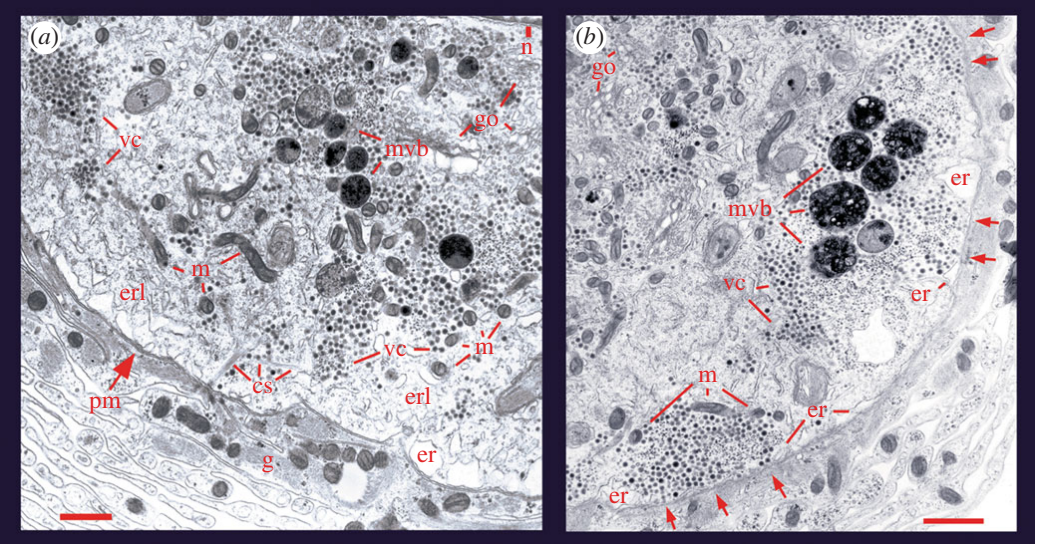
Ultrastructure of somatic release sites. (*a*) Electron micrograph of a Retzius neuron in the ganglion that had been fixed after stimulation with 1 Hz trains. The vesicle clusters (vc) remained distant from the plasma membrane (pm), although bound to it through bundles of microtubules (cs). Vesicles appear near mitochondria (m) and endoplasmic reticulum (er). The golgy apparatus (g) and the nucleus (n) are marked on the upper right side. Another population of vesicle clusters and multivesicular bodies (mvb) appear more internally. As explained later in the text, multivesicular bodies are formed after vesicle exocytosis. Retzius neurons are surrounded by layers of a giant glial cell (g). Scale bar, 500 nm. (*b*) After stimulation with 20 Hz trains, the vesicle clusters appear opposed to the plasma membrane (arrows) and flanked by endoplasmic reticulum and mitochondria. Scale bar, 1 µm. Adapted with permission from De-Miguel *et al.* [43].

The number of fluorescent spots produced per soma is determined by the frequency of trains of impulses. Ten impulses produced at 1 Hz by stimulation with a microelectrode fail to induce any exocytosis. By contrast, the same number of impulses delivered at 20 Hz promotes the formation of about 70 fluorescent spots distributed at the soma surface. As confirmed with electron micrographs, the size of each spot indicates the localized exocytosis from a cluster of vesicles [43]. Figure 1 shows micrographs obtained from the soma of neurons stimulated at 1 and 20 Hz. Micrographs obtained after stimulation at 1 Hz show clusters of vesicles resting at a distance from the plasma membrane, whereas micrographs of neurons stimulated at 20 Hz show about 50% of the vesicle clusters opposed to the plasma membrane [2]. Morphological evidence of exocytosis came from experiments stimulating exocytosis in the presence of peroxidase in the bathing solution. Peroxidase was later detected inside the vesicles by immunodetection with antibodies coupled to gold particles, thus indicating that the pattern of stimulation produces cycles of exo- and endocytosis [2].

Somatic exocytosis is not locked to stimulation, but starts after a gap of several seconds. This occurs because vesicles must be actively transported to the plasma membrane in response to stimulation [43]. Therefore, a major question resulting from these observations is how does brief electrical stimulation promote transport of vesicles to the plasma membrane and their fusion during the following minutes.

## 5. Active transport of vesicle clusters

A hint about the mechanism for the mobilization of vesicles came again from electronmicrographs. Clusters of vesicles at rest or after stimulation at 1 Hz are bound to bundles of microtubules that anchor at the plasma membrane [43]. This suggested that vesicles are carried to the plasma membrane by active transport. Electrical stimulation of neurons with 20 Hz trains in the presence of colchicine to uncouple microtubules fails to evoke any significant exocytosis, thus confirming a microtubule-dependence of the vesicle transport [43]. In addition, immunostaining and pharmacological experiments have confirmed the identities of microtubules and kinesin motors. An actin cortex and myosin motors immersed in the vesicle clusters have also been detected. These two molecular motor systems transport vesicle clusters in response to electrical activity [44].

## 6. Energy-dependence of release

The active vesicle transport requires energy in the form of ATP cleavage. An estimate of the number of ATP molecules per vesicle fused came from a combination of experimental data and a model based on diffusion and the forces of molecular motors, friction and elastic forces [43]. The model fits well with the kinetics of exocytosis, measured as the increase of the FM1–43 fluorescence spots. The premises of the model are that the latency from stimulation to the initiation of the large-scale exocytosis from any vesicle cluster depends on the distance and velocity of the vesicle transport; the dynamic interval of the fluorescence increase depends on the rate at which vesicles fuse with the membrane; and the plateau of the fluorescence increase is reached when the last vesicles in the cluster fuse and exocytosis finishes. Since exo/endocytosis from each vesicle contributes to the FM1-fluorescence with a small stepwise increase, with appropriate optics, the amplitude of the maximum fluorescence increase indicates the number of vesicles that fused. The model was fed with independent measures of the density of vesicles in the clusters and their travelling distances were measured from electronmicrographs. Our predictions are that in response to a 20 Hz stimulus train, clusters of 100 to more than 1000 vesicles are transported at about 50 nm s^−1^ for distances of less than 0.7–6.0 µm, and the rate of fusion is 0.5–7 vesicles s^−1^ [43]. The relationship between the motor forces, which depends on the travelling distances, velocities and angular force, can be transformed into the work of the vesicle transport by considering the transported mass. In this case, the work equals the Gibbs free energy (ΔG). Therefore, the number of ATP molecules cleaved per vesicle fused was calculated from the coefficient between the ΔG of the transport and the number of vesicles (*n*_ves_) multiplied by the free energy of the cleavage of an ATP molecule (ΔG_ATP_).

The predicted number of ATP molecules/vesicle decreases logarithmically as the number of vesicles in the cluster increases, thus indicating a high cooperativity in the transport process. The number of ATP molecules per vesicle is a bistable function of the resting distance of the vesicle cluster. Surprisingly, the high-energy barrier appears at a distance that equals the thickness of the actin cortex, thus suggesting that the actin cortex has a state-dependent role. At rest, the actin cortex forms a barrier for the mobilization and fusion of vesicle clusters. However, upon electrical activity, a calcium-dependent depolymerization of the actin cortex may allow vesicles to penetrate it, thus allowing the coupling of actin with the myosin that travels with the vesicle clusters. This assembles a complementary active actin–myosin transport system that propels the vesicles towards the plasma membrane at a lower energy cost [44].

## 7. Two calcium sources trigger somatic exocytosis

**Figure 2.**
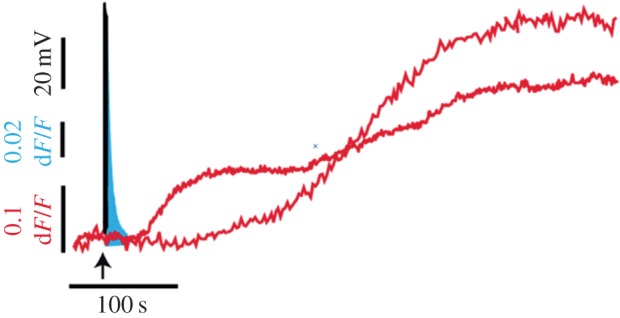
Timing of the signals that evoke somatic exocytosis. Time course of the stimulation train (black), the fast calcium transient (blue), measured as the fluorescence of Fluo-4, and exocytosis measured from the fluorescence increase of FM1–43 dye in spots produced upon fusion of vesicle clusters. The FM1–43 kinetics were obtained from different neurons. The d*F*/*F* values are the fluorescence values at each time point normalized to the baseline fluorescence. Note the logarithmic increases in the timing at each successive step.

How does the large-scale somatic exocytosis start and how is it maintained for so long after the end of electrical stimulation? In Retzius neurons, as in other neuron types of vertebrates and invertebrates, somatic exocytosis of transmitters and peptides is triggered by calcium entry through L-type calcium channels [17,45–48]. L channels are also coupled to exocytosis in gland cells [49], but not in presynaptic endings, in which P/Q or N channels predominate [50]. The slow inactivation of L channels contributes to somatic exocytosis by sustaining calcium entry during the high-frequency trains [51–53]. However, L-type calcium channels close soon after repolarization, and the calcium concentration returns rapidly to basal levels. This occurs in the soma of Retzius neurons after a 20 Hz train (that lasts 500 ms), but yet seconds before the onset of the large-scale exocytosis (figure 2). However, a 20 Hz train produces summation of the calcium transients in response to each subsequent action potential. A resulting large calcium transient reaches its peak within 600 ms and decays with a half time of 2 s [54]. Calcium entry through L-type calcium channels triggers exocytosis and blockade of these channels with nimodipine reduces the amplitude of the calcium transient by the same proportion as it reduces the amount of exocytosis [17,54]. However, somatic exocytosis requires an additional calcium source.

The fast transmembrane calcium transient activates a calcium-induced calcium release from intracellular stores. This new calcium source increases the amplitude of the fast calcium transient and the composite wave invades the whole soma (figure 2). Blockade of calcium-induced calcium release blocks somatic exocytosis by the same proportion [18,54]. By contrast, the calcium transients evoked by a 1 Hz stimulation train fail to sum up and to activate calcium-induced calcium release. Therefore, the frequency-dependence of somatic exocytosis stems from how rapidly the transmembrane calcium currents sum to increase the calcium concentration and activate calcium-induced calcium release.

## 8. A third calcium source and serotonin release sustain somatic exocytosis

A calcium- and a serotonin-dependent feedback mechanism sustain the large-scale exocytosis once the vesicle clusters arrive at the plasma membrane. This was discovered by correlating the kinetics of exocytosis (measured from the fluorescence of FM1–43 spots) with the kinetics of the intracellular calcium elevations that followed the fast transmembrane calcium transient (measured from the fluorescence of the calcium indicator Fluo-4). It is worth noting here that the high-affinity of Fluo-4 does not impair somatic exocytosis in Retzius neurons [54] in the way high affinity calcium indicators do at synapses [55].

**Figure 3.**
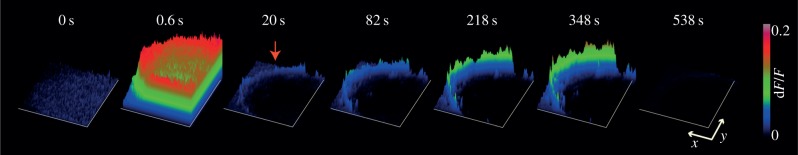
Calcium increases upon electrical stimulation in the soma of a Retzius neuron. A sequence of images made at the onset of stimulation (0 s) and at different times after stimulation show the peak of the fast calcium transient (0.6 s) invading the cell body. This transient is produced by calcium entry through L-type calcium channels and fed by calcium-induced calcium release. The contour of the cell can be inferred by the red colour. Some green and blue signals are due to out of focus light. In the following images the fast calcium transient has disappeared and instead, the peripheral serotonin-dependent calcium transient remains. The end of this transient occurs after the end of exocytosis. Adapted with permission from Leon-Pinzon *et al.* [54].

**Figure 4.**
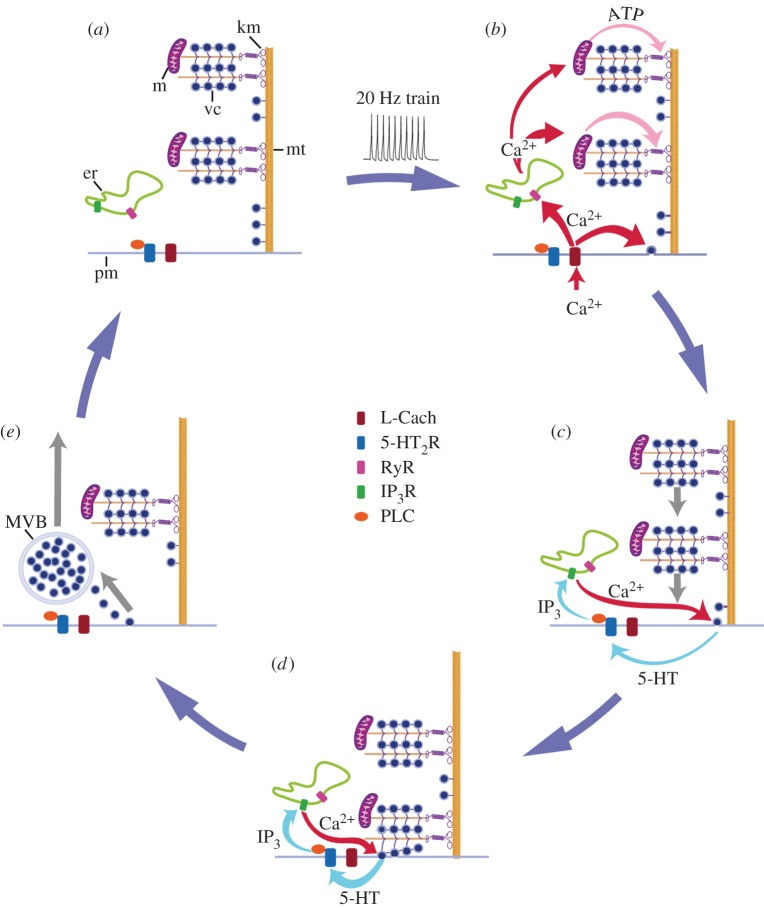
The mechanism for somatic 5-HT exocytosis by Retzius neurons. (*a*) At rest, vesicle clusters (vc) and mitochondria (m) are distant from the plasma membrane. Both are attached to microtubules (mt) that arrive at the plasma membrane (pm). Endoplasmic reticulum (er) rests between the plasma membrane and the vesicle clusters. (*b*) A train of impulses evokes transmembrane Ca^2+^ entry through L-type channels (L Cach). Ca^2+^ triggers exocytosis from vesicles that rest close to the plasma membrane and in parallel, activates ryanodine receptors (RyR) and Ca^2+^-induced Ca^2+^ release, presumably from endoplasmic reticulum. The fast Ca^2+^ transient reaches mitochondria (m), which respond by producing ATP. Kinesin motors (km) are activated. (*c*) Vesicle clusters are transported towards the plasma membrane. The peripheral vesicle clusters and mitochondria receive more Ca^2+^ and ATP than the central clusters. Therefore, they are transported more efficiently towards the plasma membrane. As vesicles arrive at the plasma membrane and fuse, the 5-HT that had been released activates 5HT_2_ receptors (5HT_2_R) coupled to phospholipase C (PLC). Activation of PLC induces the formation of IP_3_, which acts on receptors (IP_3_R) and activates Ca^2+^ release from submembrane endoplasmic reticulum (ER). Ca^2+^ evokes further exocytosis, thus closing the local feedback loop. (*d*) Arrival of vesicle clusters at the plasma membrane produces the large-scale exocytosis. (*e*) The feedback loop ends when the last vesicles in the cluster fuse. The Ca^2+^ levels return to rest and the system goes back to its off-state (*a*). Endocytosis of electrodense vesicles produces multivesicular bodies (MVB) that are transported to perinuclear regions of the soma. Image adapted with permission from Leon-Pinzon *et al.* [54].

The large-scale exocytosis is maintained by a persistent calcium elevation that remains restricted to the soma shell (figure 3). This calcium transient increases during exocytosis and returns to baseline values once exocytosis ends, thus suggesting an interlink between the intracellular calcium increase and exocytosis [54]. The peripheral calcium transient can be reproduced by iontophoretic applications of serotonin, indicating that the serotonin that had been released activates autoreceptors to produce this transient. Binding of serotonin to autoreceptors activates phospholipase-C to release intracellular calcium via inositol triphosphate (IP_3_) production (figure 4*c,d*). Consistently, somatic exocytosis and the slow peripheral calcium transient are abolished by an antagonist of serotonin receptors or by a blocker of phospholipase-C activation. However, none of these manipulations affects the triggering of the fast calcium transient locked to the 20 Hz train. These experiments confirmed that the large-scale somatic serotonin exocytosis is produced by a positive feedback loop. Calcium triggers serotonin exocytosis, and the serotonin that has been released induces an intracellular calcium increase. The feedback loop then goes from intracellular to extracellular and back. Serotonin exocytosis and the feedback loop end when the last vesicles in the cluster fuse and the intracellular calcium concentration returns to resting levels. In this way, a brief train of impulses at high frequency produces an exocytosis that may continue for hundreds of seconds. By counting the number of fluorescent spots produced per soma, and estimating the number of vesicles released per fluorescent spot from electron microscopy and modelling, we estimate that tens of thousands of serotonin quanta are released from the neuronal cell body in response to one of these trains [43,54]. While the somatic release of serotonin requires a positive feedback loop that promotes more exocytosis, the release of serotonin at synapses activates a presynaptic chloride current that inhibits the arrival of subsequent action potentials [56]. In this way, serotonin release exerts a dual auto-regulatory mechanism on its own exocytosis.

## 9. Cycling of electrodense vesicles

The differences between somatic and synaptic exocytosis of serotonin do not end with the release process. The cycling of electrodense vesicles involves another sequence of steps that differ from those of the cycling of clear vesicles at synapses [2]. After fusion, electrodense vesicles remain clustered by the membrane and gradually form multivesicular bodies (figure 4*d*). Electronmicrographs of neurons in which exocytosis was stimulated in the presence of FM1–43 or extracellular peroxidase have shown either marker inside the multivesicular bodies. Multivesicular bodies loaded with FM1–43 during the exo/endocytosis cycle can be followed for hours in living neurons during their trip back to the perikarion. Electron micrographs also show that the contents of multivesicular bodies become degraded. The multivesicular bodies release their contents upon arrival at the perikarion. This material is apparently re-used for the formation of new electrodense vesicles [2].

## 10. Where does serotonin go after being released?

‘Diffusion’ is the conventional term that has been used to describe how extrasynaptically released transmitters (or after their spill-over from synapses) reach their distant targets. However, diffusion seems to take place only in the narrow extracellular space between neurons and their surrounding glia. The major mechanism by which extrasynaptically released serotonin seems to reach its targets involves its capture and later release by the surrounding glia. As in other neuron types [57], somatic release of serotonin does not occur onto postsynaptic endings, but onto surrounding glial cells [43]. Stimulation of Retzius neurons with 20 Hz trains evokes a slow depolarization of glial cells, as recorded with an intracellular microelectrode. The time course of this depolarization resembles that for somatic exocytosis (C Trueta, JG Nicholls and FF De-Miguel 2014, unpublished data), and is too slow to be attributed to potassium liberated from the neuron [24]. The uptake of serotonin by the glia that surrounds the soma of Retzius neurons, seen with multiphoton imaging of serotonin autofluorescence, seems to occur through a sodium-dependent transport [58]. Since electron micrographs of glial cells do not show vesicles inside the glia, an attractive speculation is that they release serotonin in other regions by a reversed transporter mechanism.

## 11. The time course of the effects of extrasynaptic exocytosis of serotonin

Leeches respond experimentally to exogenous serotonin in various manners. Addition of serotonin to the water triggers swimming in leeches that have been deprived of endogenous serotonin upon injection of 5, 7-dihydroxitriptamine [59]. Moreover, intracellular stimulation of Retzius neurons with long trains of impulses increases the concentration of serotonin in the blood stream and produces fictive swimming [60].

**Figure 5.**
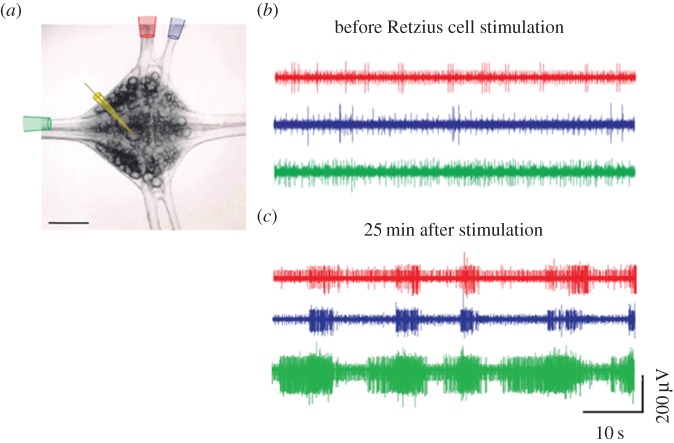
Stimulation of a Retzius neuron synchronizes the electrical activity of multiple neurons. (*a*) Intracellular stimulation with 20 Hz trains was carried out through an intracellular electrode (yellow) inserted into one of the Retzius neurons. Multiunit electrical activity was collected through suction electrodes from one connective nerve (green) and two nerve roots (red and blue). (*b*) Before one of the Retzius neurons was stimulated the electrical recordings contained non-correlated spikes produced by different neurons, as inferred from the varying unit sizes. (*c*) Twenty-five minutes after stimulation of the Retzius neuron with 20 Hz trains the electrical activity displayed a bursting pattern with multiunit synchronization.

In the experiments presented here, stimulation of Retzius neurons with trains of impulses at 20 Hz produced a delayed and long-lasting synchronization of the electrical activity of multiple neurons, as recorded with extracellular electrodes from the nerve roots and connective nerves (figure 5). In five ganglia tested, this synchronization was not locked to the stimulation trains, but appeared gradually over the 20–30 min that followed stimulation. The bursting activity appeared with an average frequency of 9.3 ± 1.2 cycles per second, being consistent with the crawling pattern obtained in semi-intact preparations [61]. This synchronization continued 2 h later, at the end of the experiments. In four preparations, the presence of the serotonin antagonist methysergide (140 µM) in the bathing fluid prevented this synchronization, and in four other preparations, the synchronous electrical activity was induced by a bath application of 10 mM serotonin. These results indicate that serotonin released from the soma (and most probably also from the axon) is, at least, the trigger for this synchronization. However, the contribution of intermediary transmitters released by the glia or other neurons in response to serotonin cannot be ruled out. In this way, a brief train of impulses is translated into a long-term modulation in the nervous system.

## 12. How is serotonin exocytosis evoked and regulated in the animal?

Retzius neurons receive multiple inputs and their firing pattern depends on which are activated. In the absence of any external stimulation, Retzius neurons fire action potentials at frequencies below 1 Hz. This firing is produced upon summation of synaptic potentials produced in the coupled dendrites of both neurons [62]. Electrical coupling between the pair of Retzius neurons contributes to this firing pattern. The pair of Retzius neurons in each ganglion is electrically coupled through a non-rectifying electrical synapse [63,64] and Retzius neurons also form electrical synapses with the five other serotonergic neurons in the ganglion [65]. The coupled dendrites of both neurons are permanently producing postsynaptic potentials upon activation of a common input [66]. By controlling the leak of synaptic currents from one coupled dendrite to another, electrical coupling regulates the amplitude of excitatory postsynaptic potentials [62]. The synaptic potentials produced in the coupled dendrites of both neurons sum in the primary axons of both neurons to produce the characteristic low firing frequency of these and other serotonergic neurons [67]. In this way, electrical coupling extends the effective dendritic tree of one neuron to that of the coupled pair, and by regulating the amount of current leakage and the amplitude of synaptic potentials, electrical coupling regulates the basal firing frequency.

The high-frequency firing of Retzius neurons that evokes somatic exocytosis can be reached by applying pressure to the skin [68]. Paired recordings from Retzius neurons and mechanosensory neurons responding to touch (T cells), pressure (P cells) or nociceptive (N cells) stimulation to the skin have shown that the non-coupled dendrites of Retzius neurons receive polysynaptic excitatory inputs from every mechanosensory neuron in the ganglion [68]. The synapse from neurons sensing touch (T neurons) in the skin depresses rapidly along a train of presynaptic impulses [68]. For this reason, activation of these inputs may evoke only a transient serotonin release from synapses without activating the large-scale somatic exocytosis. By contrast, stimulation of P neurons with trains of impulses produces facilitation of the excitatory synaptic potentials in Retzius neurons [68], and Retzius neurons are good followers of the firing frequency of pressure sensory neurons [68–70]. Pressure applied to the skin drives bursts of action potentials in Retzius neurons at frequencies above 20 Hz [68]. Therefore, this input has the characteristics required to activate the somatic exocytosis of serotonin. Activation of N cells with trains of impulses also produces facilitation of the subsequent excitatory synaptic potentials in Retzius neurons. However, owing to the low physiological firing frequency of N cells, they may only reinforce the input from P sensory neurons when the forces applied to the skin are increased [68].

## 13. General conclusion

Somatic exocytosis of serotonin is a multistep and multiregulated process. Once exocytosis is triggered, whole clusters of electrodense vesicles release their contents. In this regard, a vesicle cluster behaves as a ‘release unit’ that releases its content in an all or nothing manner. Several minutes pass between the triggering train of impulses and the end of release. A train of impulses at 20 Hz lasting for 500 ms produces a release that lasts for minutes. Release occurs onto glial cells and affects the nervous system for the following hours. Therefore, somatic release prolongs the timing of the signalling, and in this way serotonin somatic exocytosis confers a slow-onset and long-lasting timing to the modulation of hard-wired circuits. A similar timing may be responsible for our moods, and deficiencies in the extrasynaptic serotonergic release may produce deficits such as depression.

Experiments in isolated raphe neurons have shown that several steps described here may occur in mammalian neurons [13,16]. In addition, evidence in other neuron types presented in this special issue point to general mechanisms for somatic exocytosis of transmitters and peptides, in peripheral and central neurons of vertebrates and invertebrates. This may be another example of how general principles of neuronal function can be learned from an invertebrate neuron.

## 14. Material and methods

Extracellular multiunit electrical recordings from the nerve roots and connective nerves were carried out by use of glass suction electrodes made from borosilicate glass capillaries (World Precision Instruments, Germany). Glass tubes were pulled by a conventional pipette puller (P-97; Sutter Instruments Co., Novato, CA, USA). The tips were cut using a diamond knife to obtain a diameter between 80 and 150 μm. The tips were fire polished using a custom designed forge. Electrodes were filled with normal Ringer's leech solution and connected through a silver wire to P15 amplifiers. Signals were digitized at 10 kHz by an A/D converter (digidata-1200, Molecular Devices, Sunnyvale, CA, USA) and recorded and processed using pCLAMP9 software (Molecular Devices).

Retzius neurons were stimulated through an intracellular microelectrode, through which we also recorded its electrical activity during the experiment. Electrical stimulation consisted of 10 trains of 10 action potentials each, produced by 10-ms current pulses delivered at 20 Hz [17,43]. Stimulation was applied through a borosilicate microelectrode with a resistance of 18–30 M Ω when filled with 2 M potassium acetate (KAc). The amplitude of the current pulses was adjusted in every neuron between 5 and 8 nA, so that each pulse would produce an action potential. The neuronal resting potential was maintained at −60 mV by direct current injection. Data were acquired by an AxoClamp2B amplifier (Molecular Devices) connected to the same analogue-to-digital board mentioned above. Data were acquired using pCLAMP9 software (Molecular Devices) and stored in a PC.

In four other preparations, stimulation of Retzius neurons was made in the presence of the serotonin antagonist methysergide (140 µm, Sigma Aldrich). In four other ganglia, serotonin 10 mM (Sigma Aldrich) was perfused instead of applying electrical stimulation to Retzius neurons.
